# Isonicotinamide–2-naphthoic acid (1/1)

**DOI:** 10.1107/S1600536811050057

**Published:** 2011-11-25

**Authors:** Lee G. Madeley, Demetrius C. Levendis, Andreas Lemmerer

**Affiliations:** a Molecular Sciences Institute, School of Chemistry, University of the Witwatersrand, Johannesburg, PO Wits 2050, South Africa

## Abstract

In the title 1:1 adduct, C_6_H_6_N_2_O·C_11_H_8_O_2_, the amide group is slightly twisted out of the plane of the aromatic ring, with a C—C—C—N torsion angle of 25.11 (19)°, whereas the carb­oxy­lic acid group is approximately coplanar with the bicylic ring system, with a C—C—C—O torsion angle of 10.9 (2)°. The amide groups from two isonicotinamide mol­ecules form a dimer *via* N—H⋯O hydrogen bonds. In addition, the 2-naphthanoic acid mol­ecule is hydrogen bonded to the pyridine unit of an isonicotinamide mol­ecule *via* an O—H⋯N hydrogen bond. This gives rise to a centrosymmetric four-mol­ecule chain, which is cross-linked by further N—H⋯O hydrogen bonds from the amide group.

## Related literature

For related compounds, see: Lemmerer *et al.* (2008 ▶); Aakeröy *et al.* (2002 ▶); Báthori *et al.* (2010 ▶). The carb­oxy­lic acid–pyridine hydrogen bond is an often used supra­molecular synthon, see: Aakeröy & Beatty (2001 ▶). 
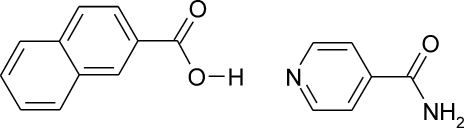


## Experimental

### 

#### Crystal data

C_6_H_6_N_2_O·C_11_H_8_O_2_
*M* *_r_* = 294.3
Monoclinic, 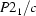

*a* = 8.6665 (17) Å
*b* = 23.752 (5) Å
*c* = 7.3793 (15) Å
β = 110.33 (3)°
*V* = 1424.4 (5) Å^3^
*Z* = 4
Mo *K*α radiation
μ = 0.10 mm^−1^
*T* = 173 K
0.48 × 0.45 × 0.08 mm

#### Data collection

Bruker APEXII CCD area-detector diffractometer
Absorption correction: integration (*XPREP*; Bruker, 2007 ▶) *T*_min_ = 0.956, *T*_max_ = 0.993
7507 measured reflections
2605 independent reflections
2130 reflections with *I* > 2σ(*I*)
*R*_int_ = 0.055

#### Refinement

*R*[*F* ^2^ > 2σ(*F* ^2^)] = 0.041
*wR*(*F* ^2^) = 0.119
*S* = 1.01
2605 reflections
211 parameters
H atoms treated by a mixture of independent and constrained refinement
Δρ_max_ = 0.21 e Å^−3^
Δρ_min_ = −0.20 e Å^−3^

### 

Data collection: *APEX2* (Bruker, 2007 ▶); cell refinement: *SAINT-Plus* (Bruker, 2007 ▶); data reduction: *SAINT-Plus* and *XPREP* (Bruker, 2007 ▶); program(s) used to solve structure: *SHELXS97* (Sheldrick, 2008 ▶); program(s) used to refine structure: *SHELXL97* (Sheldrick, 2008 ▶); molecular graphics: *ORTEP-3 for Windows* (Farrugia, 1997 ▶) and *DIAMOND* (Brandenburg, 1999 ▶); software used to prepare material for publication: *WinGX* (Farrugia, 1999 ▶) and *PLATON* (Spek, 2009 ▶).

## Supplementary Material

Crystal structure: contains datablock(s) global, I. DOI: 10.1107/S1600536811050057/fj2462sup1.cif

Structure factors: contains datablock(s) I. DOI: 10.1107/S1600536811050057/fj2462Isup2.hkl

Supplementary material file. DOI: 10.1107/S1600536811050057/fj2462Isup3.mol

Supplementary material file. DOI: 10.1107/S1600536811050057/fj2462Isup4.cml

Additional supplementary materials:  crystallographic information; 3D view; checkCIF report

## Figures and Tables

**Table 1:** Hydrogen-bond geometry (Å, °)

*D*—H⋯*A*	*D*—H	H⋯*A*	*D*⋯*A*	*D*—H⋯*A*
N1—H1*S*⋯O1^i^	0.904 (19)	2.012 (19)	2.914 (2)	176 (2)
N1—H1*A*⋯O3^ii^	0.862 (18)	2.123 (18)	2.9755 (17)	170 (2)
O2—H2⋯N2	1.05 (3)	1.56 (3)	2.5999 (18)	170 (2)

Symmetry codes: (i) 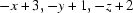; (ii) 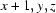.

**Fig. 1.**
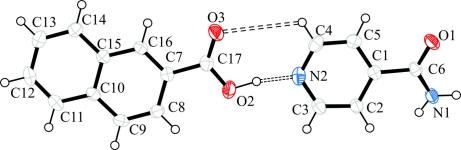
The asymmetric unit of the co-crystal showing the atomic numbering scheme. Displacement ellipsoids are shown at the 50% probability level.

**Fig. 2.**
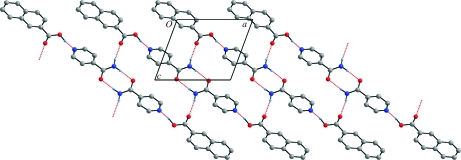
Hydrogen bonding diagram of the co-crystal. Intermolecular N—H···O and O—H···O hydrogen bonds are shown as dashed red lines forming centrosymmetric 4-molecule chains.

## supplementary crystallographic information

### Comment

This co-crystal is part of a larger crystal engineering project involving carboxylic acids and the anti-tuberculosis agent isoniazid. In this project, the pyridine N atom of either nicotinamide, isonicotinamide or isoniazid acts as a hydrogen bond acceptor for carboxylic acid group protons. The carboxylic acid-pyridine hydrogen bond is an often used supramolecular synthon (Aakeröy *et al.*, 2001; Aakeröy *et al.*, 2002; Lemmerer *et al.*, 2008). The co-crystal former ability of isonicotinamide and nicotinamide was investigated by performing density functional theory calculations in a related study (Báthori *et al.*, 2010).

The asymmetric unit of (I) consists of one molecule of isonicotinamide and one molecule of 2-naphthanoic acid, sitting on general positions (Fig. 1). The asymmetric unit is connected by a O—H···N hydrogen bond. The combination of O—H···N and N—H···O hydrogen bonds gives rise to centrosymmetric 4-molecule chains, which are cross-linked by the N—H···O hydrogen bonds (Fig. 2).

### Experimental

The compound was prepared by dissolving equimolar amounts of isonicotinamide (0.218 g) and 2-naphthanoic acid (0.308 g) in distilled methanol (15 ml). The mixture was stirred at room temperature under a standard atmosphere for 24 h. Colourless crystals were grown by slow evaporation at ambient conditions from the methanol solvent over a few days.

### Refinement

The C-bound H atoms were geometrically placed (aromatic C—H bond lengths of 0.95 Å), and refined as riding with *U*_iso_(H) = 1.2*U*_eq_(C). The N-bound and O-bound H atoms were located in the difference Fourier map and coordinates refined freely as well as their isotropic displacement parameters.

### Crystal data

**Table:**

C_6_H_6_N_2_O·C_11_H_8_O_2_	*F*(000) = 616
*M**_r_* = 294.3	*D*_x_ = 1.372 Mg m^−^^3^
Monoclinic, *P*2_1_/*c*	Mo *K*α radiation, λ = 0.71073 Å
Hall symbol: -P 2ybc	Cell parameters from 5555 reflections
*a* = 8.6665 (17) Å	θ = 1–27.5°
*b* = 23.752 (5) Å	µ = 0.10 mm^−^^1^
*c* = 7.3793 (15) Å	*T* = 173 K
β = 110.33 (3)°	Block, colourless
*V* = 1424.4 (5) Å^3^	0.48 × 0.45 × 0.08 mm
*Z* = 4	

### Data collection

**Table:**

Bruker APEXII CCD area-detector diffractometer	2130 reflections with *I* > 2σ(*I*)
ω scans	*R*_int_ = 0.055
Absorption correction: integration (*XPREP*; Bruker, 2007)	θ_max_ = 25.5°, θ_min_ = 3.0°
*T*_min_ = 0.956, *T*_max_ = 0.993	*h* = −10→10
7507 measured reflections	*k* = −28→27
2605 independent reflections	*l* = −8→7

### Refinement

**Table:**

Refinement on *F*^2^	0 restraints
Least-squares matrix: full	H atoms treated by a mixture of independent and constrained refinement
*R*[*F*^2^ > 2σ(*F*^2^)] = 0.041	*w* = 1/[σ^2^(*F*_o_^2^) + (0.0731*P*)^2^ + 0.1531*P*] where *P* = (*F*_o_^2^ + 2*F*_c_^2^)/3
*wR*(*F*^2^) = 0.119	(Δ/σ)_max_ < 0.001
*S* = 1.01	Δρ_max_ = 0.21 e Å^−^^3^
2605 reflections	Δρ_min_ = −0.20 e Å^−^^3^
211 parameters	

### Special details

**Table:**

Experimental. Numerical integration absorption corrections based on indexed crystal faces were applied using the *XPREP* routine (Bruker, 2004)
Geometry. All e.s.d.'s (except the e.s.d. in the dihedral angle between two l.s. planes) are estimated using the full covariance matrix. The cell e.s.d.'s are taken into account individually in the estimation of e.s.d.'s in distances, angles and torsion angles; correlations between e.s.d.'s in cell parameters are only used when they are defined by crystal symmetry. An approximate (isotropic) treatment of cell e.s.d.'s is used for estimating e.s.d.'s involving l.s. planes.

### Fractional atomic coordinates and isotropic or equivalent isotropic displacement parameters (Å^2^)

**Table:**

	*x*	*y*	*z*	*U*_iso_*/*U*_eq_	
C1	1.10423 (17)	0.44738 (6)	0.71685 (18)	0.0248 (3)	
C2	1.08405 (18)	0.40155 (6)	0.59333 (19)	0.0281 (3)	
H2A	1.1769	0.3829	0.5809	0.034*	
C3	0.92709 (18)	0.38371 (7)	0.48958 (19)	0.0325 (4)	
H3	0.9143	0.3518	0.4079	0.039*	
C4	0.81087 (18)	0.45238 (7)	0.6185 (2)	0.0329 (4)	
H4	0.7157	0.4703	0.627	0.039*	
C5	0.96357 (17)	0.47215 (6)	0.7311 (2)	0.0292 (3)	
H5	0.9727	0.5025	0.8179	0.035*	
C6	1.26979 (17)	0.47009 (6)	0.83740 (19)	0.0271 (3)	
N1	1.39399 (17)	0.46082 (6)	0.77495 (19)	0.0324 (3)	
H1S	1.496 (2)	0.4727 (7)	0.846 (2)	0.036 (4)*	
H1A	1.375 (2)	0.4460 (8)	0.663 (3)	0.042 (5)*	
N2	0.79178 (15)	0.40878 (6)	0.49762 (16)	0.0335 (3)	
O1	1.28451 (13)	0.49617 (5)	0.98726 (14)	0.0371 (3)	
C7	0.23811 (17)	0.35294 (6)	0.12753 (18)	0.0257 (3)	
C8	0.25613 (18)	0.30807 (6)	0.00900 (19)	0.0301 (3)	
H8	0.363	0.2962	0.0172	0.036*	
C9	0.12123 (18)	0.28196 (6)	−0.11624 (19)	0.0313 (4)	
H9	0.1354	0.2517	−0.1931	0.038*	
C10	−0.03974 (17)	0.29913 (6)	−0.13394 (19)	0.0267 (3)	
C11	−0.18280 (19)	0.27324 (7)	−0.2630 (2)	0.0350 (4)	
H11	−0.1722	0.2426	−0.3407	0.042*	
C12	−0.33562 (19)	0.29162 (7)	−0.2774 (2)	0.0382 (4)	
H12	−0.4301	0.2737	−0.3652	0.046*	
C13	−0.35493 (19)	0.33669 (7)	−0.1639 (2)	0.0355 (4)	
H13	−0.4621	0.3489	−0.1745	0.043*	
C14	−0.21997 (17)	0.36309 (6)	−0.0382 (2)	0.0289 (3)	
H14	−0.2341	0.3938	0.0371	0.035*	
C15	−0.05903 (16)	0.34512 (6)	−0.01926 (18)	0.0243 (3)	
C16	0.08343 (17)	0.37060 (6)	0.11132 (18)	0.0246 (3)	
H16	0.0717	0.4008	0.19	0.03*	
C17	0.38436 (18)	0.38080 (6)	0.2707 (2)	0.0302 (3)	
O2	0.52342 (13)	0.36681 (6)	0.25195 (16)	0.0444 (3)	
H2	0.624 (3)	0.3858 (10)	0.359 (3)	0.085 (7)*	
O3	0.37325 (13)	0.41309 (5)	0.39438 (14)	0.0404 (3)	

### Atomic displacement parameters (Å^2^)

**Table:**

	*U*^11^	*U*^22^	*U*^33^	*U*^12^	*U*^13^	*U*^23^
C1	0.0286 (8)	0.0233 (7)	0.0214 (6)	−0.0021 (6)	0.0072 (6)	0.0041 (5)
C2	0.0270 (8)	0.0301 (8)	0.0269 (7)	−0.0037 (6)	0.0091 (6)	−0.0028 (6)
C3	0.0341 (9)	0.0365 (9)	0.0271 (7)	−0.0082 (7)	0.0109 (6)	−0.0049 (6)
C4	0.0282 (8)	0.0393 (9)	0.0319 (8)	0.0055 (7)	0.0112 (6)	0.0092 (7)
C5	0.0339 (8)	0.0251 (8)	0.0288 (7)	0.0010 (6)	0.0112 (6)	0.0022 (6)
C6	0.0298 (8)	0.0236 (8)	0.0252 (7)	−0.0030 (6)	0.0061 (6)	0.0005 (6)
N1	0.0279 (7)	0.0398 (8)	0.0278 (6)	−0.0076 (6)	0.0076 (5)	−0.0106 (6)
N2	0.0276 (7)	0.0444 (9)	0.0267 (6)	−0.0059 (6)	0.0069 (5)	0.0025 (6)
O1	0.0361 (6)	0.0413 (7)	0.0340 (6)	−0.0097 (5)	0.0121 (5)	−0.0167 (5)
C7	0.0271 (8)	0.0270 (8)	0.0230 (6)	−0.0014 (6)	0.0087 (6)	0.0021 (6)
C8	0.0286 (8)	0.0304 (8)	0.0317 (7)	0.0044 (6)	0.0112 (6)	−0.0011 (6)
C9	0.0379 (9)	0.0264 (8)	0.0305 (7)	0.0026 (6)	0.0130 (7)	−0.0056 (6)
C10	0.0327 (8)	0.0213 (7)	0.0258 (7)	−0.0020 (6)	0.0098 (6)	0.0008 (6)
C11	0.0413 (9)	0.0280 (8)	0.0334 (8)	−0.0074 (7)	0.0099 (7)	−0.0056 (6)
C12	0.0316 (9)	0.0385 (10)	0.0381 (8)	−0.0129 (7)	0.0038 (7)	−0.0021 (7)
C13	0.0259 (8)	0.0379 (9)	0.0411 (8)	0.0003 (7)	0.0098 (6)	0.0079 (7)
C14	0.0301 (8)	0.0261 (8)	0.0312 (7)	0.0021 (6)	0.0115 (6)	0.0036 (6)
C15	0.0281 (8)	0.0207 (7)	0.0244 (6)	−0.0009 (6)	0.0095 (6)	0.0034 (5)
C16	0.0300 (8)	0.0206 (7)	0.0233 (7)	−0.0008 (6)	0.0093 (6)	−0.0006 (5)
C17	0.0295 (8)	0.0356 (9)	0.0249 (7)	−0.0026 (7)	0.0088 (6)	0.0009 (6)
O2	0.0242 (6)	0.0654 (9)	0.0411 (6)	−0.0041 (5)	0.0081 (5)	−0.0160 (6)
O3	0.0369 (7)	0.0517 (8)	0.0316 (6)	−0.0080 (5)	0.0107 (5)	−0.0149 (5)

### Geometric parameters (Å, °)

**Table:**

C1—C5	1.390 (2)	C8—H8	0.95
C1—C2	1.3912 (19)	C9—C10	1.415 (2)
C1—C6	1.501 (2)	C9—H9	0.95
C2—C3	1.376 (2)	C10—C11	1.415 (2)
C2—H2A	0.95	C10—C15	1.4269 (19)
C3—N2	1.335 (2)	C11—C12	1.363 (2)
C3—H3	0.95	C11—H11	0.95
C4—N2	1.339 (2)	C12—C13	1.405 (2)
C4—C5	1.378 (2)	C12—H12	0.95
C4—H4	0.95	C13—C14	1.366 (2)
C5—H5	0.95	C13—H13	0.95
C6—O1	1.2349 (16)	C14—C15	1.4182 (19)
C6—N1	1.3282 (19)	C14—H14	0.95
N1—H1S	0.904 (19)	C15—C16	1.412 (2)
N1—H1A	0.862 (18)	C16—H16	0.95
C7—C16	1.370 (2)	C17—O3	1.2215 (17)
C7—C8	1.421 (2)	C17—O2	1.3029 (18)
C7—C17	1.493 (2)	O2—H2	1.05 (3)
C8—C9	1.362 (2)		
			
C5—C1—C2	117.82 (13)	C8—C9—C10	121.18 (13)
C5—C1—C6	119.06 (13)	C8—C9—H9	119.4
C2—C1—C6	123.10 (13)	C10—C9—H9	119.4
C3—C2—C1	118.72 (14)	C11—C10—C9	122.83 (14)
C3—C2—H2A	120.6	C11—C10—C15	118.43 (13)
C1—C2—H2A	120.6	C9—C10—C15	118.73 (13)
N2—C3—C2	123.51 (14)	C12—C11—C10	120.93 (14)
N2—C3—H3	118.2	C12—C11—H11	119.5
C2—C3—H3	118.2	C10—C11—H11	119.5
N2—C4—C5	122.39 (14)	C11—C12—C13	120.75 (14)
N2—C4—H4	118.8	C11—C12—H12	119.6
C5—C4—H4	118.8	C13—C12—H12	119.6
C4—C5—C1	119.62 (14)	C14—C13—C12	120.20 (14)
C4—C5—H5	120.2	C14—C13—H13	119.9
C1—C5—H5	120.2	C12—C13—H13	119.9
O1—C6—N1	123.36 (14)	C13—C14—C15	120.63 (14)
O1—C6—C1	119.40 (13)	C13—C14—H14	119.7
N1—C6—C1	117.24 (12)	C15—C14—H14	119.7
C6—N1—H1S	119.8 (10)	C16—C15—C14	122.37 (13)
C6—N1—H1A	119.9 (12)	C16—C15—C10	118.57 (12)
H1S—N1—H1A	120.1 (15)	C14—C15—C10	119.06 (13)
C3—N2—C4	117.87 (13)	C7—C16—C15	121.72 (13)
C16—C7—C8	119.30 (13)	C7—C16—H16	119.1
C16—C7—C17	119.37 (13)	C15—C16—H16	119.1
C8—C7—C17	121.32 (13)	O3—C17—O2	123.73 (14)
C9—C8—C7	120.47 (13)	O3—C17—C7	122.62 (13)
C9—C8—H8	119.8	O2—C17—C7	113.65 (13)
C7—C8—H8	119.8	C17—O2—H2	111.5 (13)
			
C5—C1—C2—C3	1.1 (2)	C15—C10—C11—C12	−0.2 (2)
C6—C1—C2—C3	179.67 (12)	C10—C11—C12—C13	−0.2 (2)
C1—C2—C3—N2	1.5 (2)	C11—C12—C13—C14	0.6 (2)
N2—C4—C5—C1	1.7 (2)	C12—C13—C14—C15	−0.6 (2)
C2—C1—C5—C4	−2.6 (2)	C13—C14—C15—C16	−178.83 (12)
C6—C1—C5—C4	178.77 (12)	C13—C14—C15—C10	0.2 (2)
C5—C1—C6—O1	23.6 (2)	C11—C10—C15—C16	179.24 (12)
C2—C1—C6—O1	−155.01 (14)	C9—C10—C15—C16	−1.78 (19)
C5—C1—C6—N1	−156.32 (13)	C11—C10—C15—C14	0.13 (19)
C2—C1—C6—N1	25.11 (19)	C9—C10—C15—C14	179.11 (12)
C2—C3—N2—C4	−2.4 (2)	C8—C7—C16—C15	0.5 (2)
C5—C4—N2—C3	0.8 (2)	C17—C7—C16—C15	−178.93 (11)
C16—C7—C8—C9	−1.5 (2)	C14—C15—C16—C7	−179.74 (12)
C17—C7—C8—C9	177.85 (13)	C10—C15—C16—C7	1.2 (2)
C7—C8—C9—C10	0.9 (2)	C16—C7—C17—O3	11.1 (2)
C8—C9—C10—C11	179.69 (13)	C8—C7—C17—O3	−168.30 (14)
C8—C9—C10—C15	0.8 (2)	C16—C7—C17—O2	−169.70 (13)
C9—C10—C11—C12	−179.09 (14)	C8—C7—C17—O2	10.9 (2)

### Hydrogen-bond geometry (Å, °)

**Table:**

*D*—H···*A*	*D*—H	H···*A*	*D*···*A*	*D*—H···*A*
N1—H1S···O1^i^	0.904 (19)	2.012 (19)	2.914 (2)	176 (2)
N1—H1A···O3^ii^	0.862 (18)	2.123 (18)	2.9755 (17)	170 (2)
O2—H2···N2	1.05 (3)	1.56 (3)	2.5999 (18)	170 (2)

Symmetry codes: (i) −*x*+3, −*y*+1, −*z*+2; (ii) *x*+1, *y*, *z*.
